# Functional Roles of Poly(ADP-Ribose) in Stress Granule Formation and Dynamics

**DOI:** 10.3389/fcell.2021.671780

**Published:** 2021-04-26

**Authors:** Xuejiao Jin, Xiuling Cao, Shenkui Liu, Beidong Liu

**Affiliations:** ^1^State Key Laboratory of Subtropical Silviculture, School of Forestry and Biotechnology, Zhejiang A&F University, Hangzhou, China; ^2^Department of Chemistry and Molecular Biology, University of Gothenburg, Gothenburg, Sweden; ^3^Faculty of Science, Center for Large-Scale Cell-Based Screening, University of Gothenburg, Gothenburg, Sweden

**Keywords:** poly(ADP-ribose), PAR-binding, post-translational modification, stress granules, liquid–liquid phase separation

## Abstract

Stress granules (SGs) are highly dynamic cytoplasmic foci formed in response to stress. The formation of SGs is reported to be regulated by diverse post-translational protein modifications (PTMs). Among them, ADP-ribosylation is of emerging interest due to its recently identified roles in SG organization. In this review, we summarized the latest advances on the roles of poly(ADP-ribose) (PAR) in the regulation of SG formation and dynamics, including its function in modulating nucleocytoplasmic trafficking and SG recruitment of SG components, as well as its effects on protein phase separation behavior. Moreover, the functional role of PAR chain diversity on dynamic of SG composition is also introduced. Potential future developments on investigating global ADP-ribosylation networks, individual roles of different PARPs, and interactions between ADP-ribosylation and other PTMs in SGs are also discussed.

## Introduction

Stress granules (SGs) are cytoplasmic membraneless structures that rapidly assemble in cells in response to a variety of stresses, including heat, oxidative stress, and virus infection (Buchan and Parker, 2009). These liquid-like, higher-ordered condensates consist of stalled untranslated mRNA, ribosome proteins, initiation factors, diverse RNA-binding proteins (RBPs) and non-RBPs (Kedersha et al., 2005). Formation of SGs enhances cell survival by protecting certain RNAs and proteins from decay upon exposure to adverse environmental conditions, as well as by regulating intracellular signal transduction to overcome detrimental environmental conditions. As soon as the stress is relieved, SGs are disassembled to allow the mRNAs back into the translation machinery so that protein synthesis can be rapidly re-initiated (Kedersha et al., 2013; Cao et al., 2020). Continuous formation of SGs or disrupted disassembly of SGs results in aberrant conversion of SG into a pathogenic state. This type of pathogenic state is associated with a barrage of diseases, including amyotrophic lateral sclerosis (ALS), Alzheimer’s disease, and Parkinson’s disease (Alberti and Dormann, 2019; Falahati and Haji-Akbari, 2019). Thus, precise modulation of SG dynamics is critical to the maintenance of normal physiological functioning of cells. Previous studies have reported that protein post-translational modification (PTM) is one of the mechanisms by which cells control the assembly and disassembly of SGs (Buchan and Parker, 2009; Cao et al., 2020). In this mini-review, we focus on how ADP-ribosylation, an important PTM, participates in the regulation of the phase behavior of SG components and influences the formation and dynamics of SGs.

## ADP-Ribosylation

ADP-Ribosylation is a conserved, reversible PTM of proteins best known for its function in a multitude of cellular processes, such as stress response, DNA repair, signal transduction, and apoptosis (Corda and Di Girolamo, 2003; Perina et al., 2014; Bai, 2015; Qi et al., 2019). This PTM regulates protein functions via two manners: covalent modification of substrates and ADP-ribose-mediated non-covalent association with substrates (Grimaldi et al., 2019). The first relies on the covalent transfer of ADP-ribose (one or more units) to targets; namely, mono(ADP-ribosylation) (MARylation) or PARylation (Leung, 2020). Alternatively, some proteins can non-covalently bind to MARylated or PARylated targets through ADP-ribose-binding domains. This ADP-ribose-binding property is also important for their functional regulation (Krietsch et al., 2013; Cheruiyot et al., 2015; Zhang et al., 2019). PAR formation is catalyzed by a family of PAR polymerases (PARPs) (Otto et al., 2005). Some PARPs function to transfer a single ADP-ribose onto the target, while others have the capacity to add subsequent ADP-ribose units to extend MARylation (Vyas et al., 2014). The reverse process is fulfilled by PAR glycohydrolase (PARG), which cleaves the ribose-ribose bond and removes the ADPr unit from the PAR chain (Slade et al., 2011). However, this enzyme cannot remove the terminal ADP-ribose; the final ADP-ribose on the target needs to be hydrolyzed by several degraders. There are three macrodomain-containing proteins, MacroD1, MacroD2, and TARG1, that can specifically remove the terminal ADPr on Glu and Asp. In addition, ARH1 (Arg specific) and ARH3 (both PAR chains and Ser specific for the terminal ADPr) are also relevant (Jankevicius et al., 2013; Rosenthal et al., 2013; Sharifi et al., 2013). PARylated proteins can be covalently modified on different amino acids, including Glu, Asp, Arg, Ser, Cys, and Tyr (Gupte et al., 2017; Palazzo et al., 2017; Crawford et al., 2018; Leslie Pedrioli et al., 2018; Luscher et al., 2018; O’Sullivan et al., 2019). PAR-binding proteins can bind to free PAR or PARylated proteins via multiple domains, including macrodomain, PAR-binding motif (PBM), poly(ADP-ribose)-binding zinc-finger (PBZ) domain, tryptophan-tryptophan-glutamate (WWE) domain, oligosaccharide-binding fold domain (OB fold), RNA recognition motif (RRM), arginine-glycine-glycine motif (RGG), PilT N-terminus (PIN) domain, and WD40 domain (Grimaldi et al., 2019). For example, macrodomains are evolutionarily conserved domains which have high-affinity to ADP-ribose, which usually bind to mono-ADP-ribose and the terminal ADP-ribose (Karras et al., 2005; Ahel et al., 2009). PBM is enriched of basic and hydrophobic amino acids, and the positively charged residues in this domain could provide favorable electrostatic interactions with PAR (Pleschke et al., 2000; Fahrer et al., 2007). PBM-containing protein, Werner syndrome protein, can bind to PAR to regulate its enzymatic activities (Popp et al., 2013). PBZ domains usually recognize adjacent ADP-ribose groups through binding to adenines in two adjacent ADP-ribose units while WWE domain binds to *iso*-ADP-ribose (Li et al., 2010; Wang et al., 2012; Leung, 2017). Several PARPs possess WWE domains, such as PARP12. It requires its first WWE domain to bind ADP-ribose to regulate its translocation (Catara et al., 2017). It has also been found that the DNA-binding motif OB fold binds to PAR. OB fold-containing human ssDNA-binding protein 1 (hSSB1) has a high affinity with *iso*-ADP-ribose and this binding promote its recruitment to DNA damage sites (Zhang et al., 2014). RRM and RGG are abundant protein domains in eukaryotes, and many proteins containing these domains are involved in SG assembly, such as G3BP and FUS. They bind to PAR to regulate their functions (Isabelle et al., 2012; Mastrocola et al., 2013). Detailed structural features and more examples of proteins with these specialized binding domains have been extensively reviewed by Grimaldi et al. (2019).

## Poly(ADP-Ribose) Regulates SG Formation and Dynamics Through Diverse Mechanisms

Recent studies have revealed that PAR functions in the control of SG assembly and disassembly. One interesting example is that the non-structural protein 3 (nsP3) of alphaviruses can remove the PARylation modification from the SG component G3BP1 and thus inhibit SG formation. This ability depends on the conserved macrodomain of nsP3 which function as a mono-ADP-ribosylhydrolase. Therefore, it is more likely that the nsP3 macrodomain prevents MARylation and subsequent indirectly PARylation, instead of PAR chain degradation (Eckei et al., 2017; Jayabalan et al., 2021). The role of PAR in SG regulation is also supported by the following evidence: Several PARPs, including PAR-adding PARP5a, MAR-adding PARP12, PARP14, and PARP15, inactive PARP13.1 and PARP13.2, and two PARGs, PARG99 and PARG102, are localized to cytoplasmic SGs (Leung et al., 2011; Isabelle et al., 2012; Catara et al., 2017). Moreover, overexpression of PARPs, including both MAP-adding and PAR-adding PARPs, can induce the formation of SGs (Leung et al., 2011). Along the same line of evidence, overexpression of PARGs can prevent SG formation. Furthermore, accumulation of PAR in the cell, caused by the absence of PARGs, delays disassembly of SGs (Leung et al., 2011; Leung, 2014; Catara et al., 2017). These evidences indicate that PAR, the product synergistically synthesized by these enzymes, plays a critical role in normal SG assembly, dynamics, and disassembly.

**FIGURE 1:**
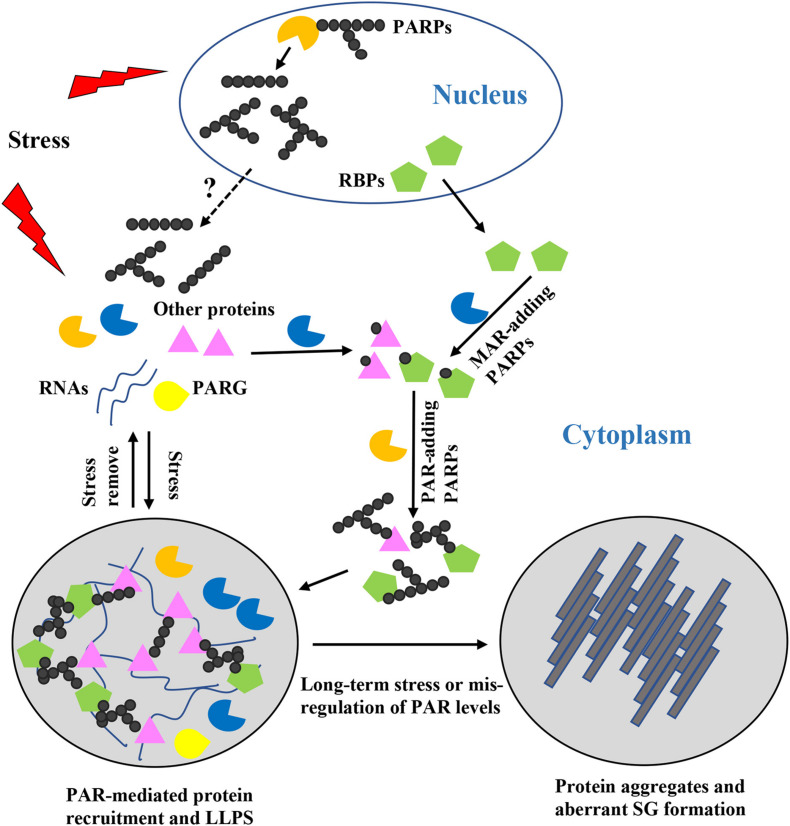
Roles of poly(ADP-ribose) (PAR) in stress granules (SG) formation, composition, and dynamics. When cells are exposed to stress, nuclear and cytoplasmic PARPs are activated, resulting in increased PAR chains with different lengths and structures. But how the PAR chains shuttle between the nucleus and the cytoplasm remains unclear. Formation of PAR chains is mediated through MAR, which might be a rate-limiting step. MAR-adding PARPs may act synergistically with PAR-adding PARPs to regulate stress granule formation. PARylated proteins or proteins bound to PARylated substrates are recruited to the specific sites where SG formation occurs and where they induce liquid-liquid phase separation. Diverse PAR chains contribute to diverse protein-protein and protein-RNA interactions. Once the stress is removed, PAR is degraded by PARG and the SGs are disassembled. However, if cells are exposed to long-term stress or PAR levels are mis-regulated, phase separated proteins are converted into protein aggregates and aberrant pathogenic SGs are formed.

How does PAR regulate these SG related-processes? Recent in-depth studies have shown that PAR controls SG formation and dynamics via different mechanisms (Figure 1). The effects of PAR on SG components are summarized in Table 1.

**TABLE 1:** Effects of poly(ADP-ribose) (PAR) on stress granules (SG) components.

PARPs	SG component	Manner	Effects	References
PARP1	hnRNP A1	PARylation at K298, binding to PAR through PAR-binding motif	Nucleocytoplasmic transport, SG association, LLPS induction, and co-LLPS with TDP-43	Duan et al., 2019
Tankyrase/PARP5	TDP-43	Binding to PAR through PAR-binding motif	SG association, LLPS induction, and inhibition of aggregate formation	McGurk et al., 2018a,b
	G3BP	Binding to PAR through C-terminal glycine-arginine-rich region	SG formation	Isabelle et al., 2012
PARP1	PARP12	Binding to PAR through WWE domain	SG association	Catara et al., 2017
	FUS	Binding to PAR through RGG motif	LLPS induction, aggregate formation	Altmeyer et al., 2015; Patel et al., 2015
	TIA-1	PARylation in RRM?		Leung et al., 2011
	Ago2	PARylation in PIWI domain?		Leung et al., 2011

### Poly(ADP-Ribose) Regulates Proteins Targeting to SGs

Several studies have found that PAR is present in SGs, and that many proteins in SGs are PARylated or possess PAR-binding domains, such as heterogeneous nuclear ribonucleoprotein A1 (hnRNP A1), TAR DNA binding protein 43 kDa (TDP-43), Ras-GTPase activating protein SH3 domain-binding protein (G3BP), argonaute family member Ago2, and T-cell intracellular antigen-1 (TIA-1) (Leung et al., 2011; Isabelle et al., 2012). For example, RNA-binding protein G3BP is a tunable switch that triggers RNA-dependent liquid-liquid phase separation and SG assembly (Guillén-Boixet et al., 2020; Yang et al., 2020). A recent study found that PAR binds to the G3BP C-terminal glycine-arginine-rich domain via non-covalent PAR binding. Such binding allows G3BP to maintain cytoplasmic localization and subsequent formation of SGs (Table 1; Isabelle et al., 2012). Moreover, PAR is required for PARP12 re-localization to SGs. Upon exposure to stress, nuclear PARP1 is activated and synthetic PAR polymers bind to the first WWE domain of PARP12, contributing to the translocation of PARP12 from the Golgi complex to SGs. This leads to the disassembly of Golgi membranes and blockage of anterograde-membrane traffic (Table 1; Catara et al., 2017). But so far, there is no clear evidence showing how the PAR chain is generated in the cytoplasm or whether the PAR chain is released from the nucleus. Translocation of TDP-43 is also PAR-dependent. Downregulation of a PARP in *Drosophila* reduces TDP-43 cytoplasmic location, and in mammalian cells and neurons, PAR- or PAR-scaffold-binding through PBM is required for TDP-43 accumulation in cytoplasmic SGs (Table 1; McGurk et al., 2018a). hnRNP A1 is another predominantly nuclear protein that can translocated to the cytoplasm upon exposure to stress (Geuens et al., 2016; Mohagheghi et al., 2016). It can be PARylated, and can also bind to PAR or PARylated proteins. Inhibition of PARP or PARG affects hnRNP A1 recruitment to or retrieval from SGs, indicating that PAR is critical for hnRNP A1’s SG location. Further analysis shows that PARylation and PAR-binding have different functions, as PARylation on K298 of hnRNP A1 is required for its cytoplasmic trafficking, while association with PAR or PARylated targets controls its sorting to SGs (Table 1; Duan et al., 2019). These studies provide strong support for the importance of PAR in regulating proteins targeting to SGs (Figure 1).

### Poly(ADP-Ribose) Affects Protein Phase Separation Behavior of SG Components

It has been reported that SG formation is induced by liquid-liquid phase separation (LLPS) of intrinsically disordered proteins (IDPs) and RNAs (Hyman et al., 2014; Gomes and Shorter, 2019). IDPs lack defined 3D structures and are highly flexible, which allows diverse, promiscuous interactions that drive LLPS formation and protein condensation (Deiana et al., 2019). And a previous study demonstrated that certain RNAs can act as seeds that initiate phase separation and FUS-containing assembly formation (Schwartz et al., 2013). Recently, a new mechanism has arisen for this seeding event. Several groups have shown that PAR, a nucleic acid-mimicking biopolymer, can stimulate liquid demixing of IDPs or proteins with low-complexity regions *in vitro* or *in vivo*. For instance, a recent publication on apoptosis signal-regulating kinase 3 (ASK3) indicated that PAR could keep ASK3 condensates in the liquid phase and enable cells to sense osmotic stress (Watanabe et al., 2021). LLPS of SG components including fused in sarcoma (FUS), TDP-43, and hnRNP A1 are also regulated by PAR (Altmeyer et al., 2015; Patel et al., 2015; Kam et al., 2018; McGurk et al., 2018a; Duan et al., 2019; Singatulina et al., 2019). For instance, *in vitro* addition of PAR polymers promotes hnRNP A1 LLPS (Table 1). And the positive effect of PAR on phase separation of hnRNP A1 was not observed in a PAR-binding-deficient mutant, demonstrating that PAR-binding ability is required for this process. In addition, PAR-binding also regulates interactions between hnRNP A1 and TDP-43, and *in vitro* assays have shown that PAR addition enhances co-LLPS of these two proteins (Table 1; Duan et al., 2019). Moreover, PAR not only elevates LLPS of TDP-43, but also mitigates TDP-43 granulo-filamentous aggregation, which is predominantly found during disease (Table 1; McGurk et al., 2018b). Thus, association with PARylated scaffolds or PAR chains not only ensures the proper targeting of proteins to SGs, but also facilitates their LLPS (Figure 1; Duan et al., 2019).

It has been observed that PAR levels can affect protein phase separation behavior McGurk et al., 2018a; Duan et al., 2019). PAR levels are strictly regulated in cells by PARPs and PARGs, and their chain length varies from 2 to 200 ADP-ribose units. Therefore, PAR chains may serve as multivalent platforms for non-covalent binding of proteins (Leung, 2020). Upon exposure to stress, PARPs are activated and local PAR levels are rapidly increased; thus, PBM-containing proteins can sense the concentrated PAR and are recruited to specific sites. This is possibly why PARPs are included in SGs and PAR-binding ability is important for components anchoring to SGs (McGurk et al., 2018a; Duan et al., 2019). The longer the PAR chain and the more binding sites it provides, the more abundant the proteins are recruited and bind to the chain. Similarly, if a PAR is covalently conjugated on different amino acids of a single protein, it is possible that this covalently modified protein can create a scaffold for other PAR-binding proteins to increase the local concentration of macromolecules. Once the concentration exceeds threshold, LLPS starts and a membraneless condensate will be formed (Molliex et al., 2015; Cao et al., 2020). This is consistent with the fact that the addition of PAR promotes TDP-43 and hnRNP A1 LLPS in a dose-dependent manner (McGurk et al., 2018a; Duan et al., 2019). If two or more factors that initiate SG formation can interact with each other and be covalently modified at the same stage, they would provide a larger platform for subsequent interaction network formation. Since the PARylation of certain proteins depends on their PAR-binding ability (Fischbach et al., 2018; Kim et al., 2019), covalent modification events may initially occur and serve as triggers for the recruitment of PAR-binding proteins. Subsequently, these PAR-binding proteins are further covalently modified to finally form a complex protein-PAR interaction network. Once the stress is removed, PAR is degraded by PARG and higher-ordered condensates are disassembled (Figure 1). PAR can be produced in a short time in the nucleus, usually from seconds to minutes. For example, upon DNA damage, PARPs are recruited within seconds and PAR are rapidly synthesized at the DNA damage sites to repair DNA (Gupte et al., 2017; Palazzo et al., 2017; Luscher et al., 2018; O’Sullivan et al., 2019). It might give the cell an ability to respond quickly to environmental changes. However, little information is available concerning the PAR chain formation in SG. It might take longer time than the synthesis in the nucleus but this needs to be further investigated.

Mutations of RBPs such as TDP-43 and hnRNP A1 have been identified as associated with many neurodegenerative diseases, including ALS, frontotemporal degeneration (FTD), and myopathy. This has been correlated with the aggregation-prone propensities of these mutants in the cytoplasm, resulting in SGs nucleation and pathological SGs formation (Sreedharan et al., 2008; Kim et al., 2013; Prasad et al., 2019). Although PAR can promote LLPS, it is worth noting that when PAR levels increase in an *in vitro* phase separation system, the dynamics of the hnRNP A1 droplet are reduced (Duan et al., 2019). Moreover, PAR can accelerate the formation of pathogenic solid phase aggregates of an ALS-linked mutation of TDP-43 that is defective in LLPS ability (McGurk et al., 2018a). Other groups found that long-term incubation of RBPs with PAR accelerates the formation of disease-related solid phase aggregates (Altmeyer et al., 2015; Kam et al., 2018). The above-mentioned proteins are known to contain disordered domains, and these disordered domains can also cause them to be more aggregation-prone (Alberti et al., 2009). Therefore, in the early stage, PAR may have the ability to initiate and accelerate LLPS. But with time, if PAR continues to exist in high concentrations or under disease conditions, it may enhance the intrinsic aggregation propensity of proteins especially disease-associated mutants (Altmeyer et al., 2015; McGurk et al., 2018a; Duan et al., 2019; Liu and Fang, 2019). Hyperactivation of PARP and/or increased levels of PARylation have been found in patients or animal models of some neurodegenerative diseases (Liu and Fang, 2019; McGurk et al., 2019). Under long-term stress or disease conditions, when PARylation and PAR levels are dysregulated, PAR has the tendency to alter protein phase separation behavior and further develop into solid protein aggregates, which may result in pathological SG formation (Figure 1). This is supported by the observation of decreased PARylation levels following PARP knockdown or treatment with PARP inhibitor can antagonize cytoplasmic aggregation of the disease-related proteins hnRNP A1 and TDP-43 and their mediated neurotoxicity (Duan et al., 2019).

Taken together, PAR can serve as a scaffold for inducing LLPS of macromolecules and SG assembly. However, when PARP activity and PAR levels are mis-regulated, the proteins and granules may condense into irreversible solid phase aggregations that contribute to neuropathies (Figure 1).

### Poly(ADP-Ribose) Regulates the Dynamic of SG Composition

The PAR chain can vary from a few to hundreds of ADP-ribose units (Leung, 2020). Studies suggested that different proteins have different PAR chain length preference. Some proteins prefer long PAR chains while other proteins bind to short chains more efficiently (Fahrer et al., 2007, 2010; Popp et al., 2013). Meanwhile, proteins containing different PAR-binding domains can associate with different types of ADP-ribose groups. For example, terminal ADP-ribose units are recognized by macrodomains while adjacent ADP-ribose units are recognized by the PBZ domain (Li et al., 2010; Leung, 2017). Therefore, the PAR chain provides the possibility to recruit diverse proteins in one place, and this ability is enzymatically controlled through regulating polymer size and structures (linear or branched) in a precise temporal order (Figure 1). SGs are highly dynamic and include different components that are utilized to respond to different types of stress. During the SG formation process, the composition of an SG may vary dramatically (Buchan and Parker, 2009; Protter and Parker, 2016). Distinct PARPs and PARGs resident in SGs function in the recognition of their individual targets–either to pull them together into the SG or to exclude unwanted proteins from the SG, according to the cell’s needs. Also, as many PAR-binding proteins are RBPs, which have high affinities for RNA, PAR may compete with RNA for protein binding (Leung, 2020). Therefore, covalent PAR formation on targets and PAR-binding contribute to diverse protein-protein interactions and protein-RNA interactions, providing another means of regulation of SG formation and controlling of SG components’ dynamic.

## Conclusion and Perspective

Poly(ADP-ribose) has become an emerging research topic due to its recently identified roles in the organization of SGs and pathogenesis of SG-related neurodegenerative diseases (Grimaldi et al., 2019; McGurk et al., 2019). We summarized the possible mechanisms by which PAR mediates SG formation and dynamics as follows. PAR can promote the proper localization of SG components to SGs through ADP-ribosylation or PAR-binding, and this PTM assists in protein LLPS and SG formation. Various PAR chain lengths and structures could help in diverse SG composition. More importantly, when PAR levels are mis-regulated, the physical properties of SGs may become altered, and aberrant liquid-solid phase separation and pathogenic SGs may form (Figure 1). Besides these investigations on the roles of PAR in localizing SG components and regulating their LLPS behaviors, mechanisms underlying the interplay between PAR and SGs under different types of stress, individual roles of various PARPs in SGs, and the links between ADP-ribosylation and other PTMs remain unclear.

Further in-depth investigations are needed to clarify the interplay between PAR and SGs under different conditions. Such as what are the PAR-mediated interaction networks in SGs. This includes systematically determination of SG components that are PARylated or PAR-bound under different stresses. Different groups have used diverse approaches to identify PARylated vs. PAR-binding proteins (Daniels et al., 2015; Vivelo and Leung, 2015; Bonfiglio et al., 2017; Ando et al., 2019; Dasovich et al., 2021), which could be used as valuable tools on deciphering which SG components are PARylated and whether they undergo the same ADP-ribosylation modifications under different types of stress. For example, in a recently published paper, hundreds of novel PAR-binding proteins were identified using photoaffinity-based proteomics, which provides us with a valuable resource for exploring proteins involved in LLPS and SG formation (Dasovich et al., 2021). Moreover, innovative approaches are needed to systematically identify the modified sites on SG components and even the PAR chain length and structure on each site. It would be beneficial for identifying SG scaffold proteins and their binding partners, and building the overall PAR-mediated interaction networks in SGs. However, there are studies that failed to identify PARPs in SGs (Markmiller et al., 2018; Youn et al., 2018). Therefore, whether the initiation and dynamic maintenance of diverse SGs absolutely require PAR? Up to now, the mechanism of SG formation is not fully resolved, and the diversity of SG is stress or cell-type dependent. Does SG formation under different stress conditions have different requirements for PAR? Systematic analysis on the involvement of PARPs/PAR in SG under different conditions using different models will provide a global view on the importance of PAR in SG formation, and such systematic studies will increase our understanding on the formation mechanisms of SGs.

The other remaining question is what are the individual roles played by various PARPs in SGs, and whether or not they affect each other. Six reported SG-localized PARPs might have their own specific targets and functions. They might specifically regulate the localization, LLPS behavior, and interaction network of their substrates. For example, previous study indicated that long-term stress result in excessive cytoplasmic accumulation of the disease-related protein TDP-43, and reduction of PARP5a/5b activity could antagonize cytoplasmic aggregation of TDP-43 and its mediated neurotoxicity (McGurk et al., 2018a). Therefore, determination of which PARP responsible for the specific modification of different SG components could help in the development of novel therapeutic avenues, which could specifically regulate the formation and composition of pathogenic SGs.

Moreover, what are the links between ADP-ribosylation and other PTMs. Do they work together synergistically or compete with each other on substrates via conjunction on the same or adjacent motifs in SG regulation? A previous study indicated that PAR-dependent anchoring of TDP-43 to SGs inhibits its disease-associated phosphorylation. If TDP-43 were to accumulate excessively in the cytoplasm and become excluded from SGs, it could become phosphorylated and form irreversible aggregates (McGurk et al., 2018a). Therefore, as a single SG component may be modified by multiple PTMs, there might be interplays between ADP-ribosylation and other PTMs. PAR synthesis and degradation is a highly dynamic process, whose timely regulation ensures proper formation and dynamics of SGs. But how do cells finely regulate these processes? Should other PTMs such as phosphorylation block continued ADP-ribosylation on same substrate to ensure that the high levels of PAR are transient, preventing PARylated or PAR-binding proteins from transformation into pathogenic aggregates under long-term stress. Thus, more details of the mechanism of interplay between ADP-ribosylation and other PTMs need to be revealed.

Poly(ADP-ribose) as an important novel regulator of SG formation and dynamics, in-depth investigations on the remaining questions discussed above will help us to acquire a clear mechanistic view on global PAR-mediated interaction networks, specific functions of PARPs and links between ADP-ribosylation and other PTMs in SGs. This will eventually contribute on the development of novel therapeutic approaches targeting the aberrant pathogenic SG formation and further pave the way for effective SG-related neurodegenerative disease treatments.

## Author Contributions

XJ, XC, and BL contributed to the design and wrote the manuscript. BL and SL supervised the overall direction, planning, and editing of the manuscript. All authors contributed to the article and approved the submitted version.

## Conflict of Interest

The authors declare that the research was conducted in the absence of any commercial or financial relationships that could be construed as a potential conflict of interest.
